# A Delphinidin-Enriched Maqui Berry Extract Improves Bone Metabolism and Protects against Bone Loss in Osteopenic Mouse Models

**DOI:** 10.3390/antiox8090386

**Published:** 2019-09-10

**Authors:** Masahiro Nagaoka, Toyonobu Maeda, Masahiro Chatani, Kazuaki Handa, Tomoyuki Yamakawa, Shuichi Kiyohara, Takako Negishi-Koga, Yasumasa Kato, Masamichi Takami, Shumpei Niida, Stefanie C. Lang, Marlena C. Kruger, Keiko Suzuki

**Affiliations:** 1 Department of Pharmacology, School of Dentistry, Ohu University, Fukushima 963-8611, Japan; 2 Department of Oral Function and Molecular Biology, School of Dentistry, Ohu University, Fukushima 963-8611, Japan; 3 Department of Pharmacology, School of Dentistry, Showa University, Tokyo 142-8551, Japan; 4 Medical Genome Center, National Center for Geriatrics and Gerontology (NCGG), Aichi 474-8511, Japan; 5 Anklam Extrakt GmbH, Marienbergstr. 92, 90411 Nuremberg, Germany; 6 School of Health Sciences, College of Health, Massey University, Palmerston North 4442, New Zealand

**Keywords:** maqui berry, delphinidin, anthocyanin, oxidative stress, antioxidant, osteoblast, osteoclast, NF-κB, osteopenic mouse

## Abstract

In our previous investigation, delphinidin, one of the most abundant anthocyanins found in vegetables and berry fruits, had been shown to inhibit osteoclasts and prevent bone loss in mouse models of osteoporosis. In the present study, we investigated whether a delphinidin glycoside-enriched maqui berry extract (MBE, Delphinol^®^) exhibits beneficial effects on bone metabolism both in vitro and in vivo. MBE stimulated the osteoblastic differentiation of MC3T3-E1 cells, as indicated by enhanced mineralized nodule formation, and increased alkaline phosphatase activity, through the upregulation of bone morphogenetic protein 2 (*Bmp2*), runt-related transcription factor 2 (*Runx2*), osterix (*Osx*), osteocalcin (*Ocn*), and matrix extracellular phosphoglycoprotein (*Mepe*) mRNA expression. Immunostaining and immunoprecipitation assays demonstrated that MBE suppressed NF-κB transnucleation through acting as a superoxide anion/peroxynitrite scavenger in MC3T3-E1 cells. Simultaneously, MBE inhibited both osteoclastogenesis in primary bone marrow macrophages and pit formation by maturated osteoclasts on dentine slices. Microcomputed tomography (micro-CT) and bone histomorphometry analyses of femurs demonstrated that the daily ingestion of MBE significantly increased BV/TV (ratio of bone volume to tissue volume), Tb.Th (trabecular thickness), Tb.N (trabecular number), N.Nd/N.Tm (node to terminus ratio), OV/TV (ratio of osteoid volume to tissue volume), BFR/TV (bone formation rate per tissue volume), and significantly decreased Tb.Sp (trabecular separation), ES/BS (ratio of eroded surface to bone surface) and N.Oc/BS (number of osteoclast per unit of bone surface), compared to vehicle controls in osteopenic mouse models. These findings suggest that MBE can be a promising natural agent for the prevention of bone loss in osteopenic conditions by not only inhibiting bone resorption, but also stimulating bone formation.

## 1. Introduction

Osteoporosis is a common disease characterized by decreased bone mass and impairment of the skeletal architecture, resulting in an increased frequency of fracture. Osteoporosis occurs in more than 30% of postmenopausal women [1], and is strongly associated with diminishing estrogen levels. It is also well recognized that a decline in estrogen levels impacts on the differentiation and activity of osteoclasts (OC) and osteoblasts (OB), thus enhancing the rate of activation and resulting in an imbalance between bone resorption and bone formation, leading to decreased bone mass [2]. Alternatively, several studies have reported that estrogen deficiency caused bone loss by decreasing thiol antioxidants in OC, indicating that estrogen exerted beneficial effects by suppressing reactive oxygen species (ROS) in bones, similar to in many other tissues [3,4,5]. In addition to this, increased oxidative stress has been shown to be the key mechanism leading to bone loss seen in the elderly, in both men and women [6,7,8]. Thus, osteoporosis is now known to be one of the chronic lifestyle-related diseases.

In order to maintain bone health and ensure mobility, even after longitudinal bone growth stops, adequate amounts of new bone need to be formed following bone resorption [9]. Further, it is well established that ROS production contributes to the stimulation of bone resorption [10,11], whereas OB differentiation is inhibited by ROS [11,12]. In a review by Hubert assessing the impact of polyphenols derived from berries on bone density, it was concluded that high berry consumption was significantly correlated with increased bone mass [13]. The inclusion of antioxidants, which can scavenge ROS, could be important in the prevention of bone loss before menopause, and this may result in a reduced risk of fractures with aging.

Natural compounds such as polyphenols and other antioxidants found in fruits and vegetables have several health benefits, including a role in the prevention of bone loss. Some types of flavonoids, such as hesperidin, quercetin, and luteolin, have been reported to protect against bone loss in the ovariectomized mouse model [14,15,16]. Likewise, an anthocyanin-rich phytochemical from blueberries prevented bone loss in an ovariectomized rat model [17]. Anthocyanins, a family of flavonoids found in berry fruits and vegetables, can provide numerous beneficial effects to humans, including bone health [18,19,20,21], most likely through their antioxidant activity [20]. Research has shown that anthocyanins have stronger antioxidant properties compared to other flavonoids. This may be due to their charged oxygen atom, i.e., flavylium cation [22], and their ability to eliminate free radicals by donating a hydrogen atom from its hydroxyl group. In fact, maqui berries (*Aristotelia chilensis*), up to now the richest natural source of anthocyanins [23,24] with high antioxidant activity [24], have been described as possessing a wide range of remarkable nutritional and health-promoting features for humans [25,26,27].

In addition, anthocyanins derived from blackcurrant and bilberry significantly inhibited NF-κB activation in monocytes, resulting in the downregulation of proinflammatory mediators [28,29]. Lean et al. reported that ovariectomy-induced bone loss, in which TNFα expression was upregulated, was abolished by antioxidants, which increased tissue glutathione levels [3]. Furthermore, it is well established that the NF-κB signaling pathway is critical for osteoclastogenesis. Consistent with this evidence, our previous data showed that purified delphinidin blocked osteoclastogenesis by inhibiting the transnucleation of the p65 subunit of NF-κB [30].

In contrast, NF-κB is also shown to inhibit OB differentiation and bone formation in vivo [12,31,32,33]. The activation of OB, together with the inhibition of OC, is important to maintain the bone structure, as OB are responsible for bone matrix protein production and mineralization. Considering that ROS can cause NF-κB activation, delphinidin, one of the phytochemicals possessing antioxidant activity, may attenuate NF-κB transnucleation in OB, and thereby restore bone formation. Likewise, several studies revealed that peroxynitrite, produced by reacting nitric oxide with superoxide, caused the tyrosine nitration of NF-κB inhibitor (I-κB), which in turn increased NF-κB nuclear translocation, resulting in cell death in various cell types [34,35,36,37]. Additionally, Alund et al. observed that N-acetyl cysteine, one of the dietary antioxidants, partially prevented alcohol-induced osteopenia in mice by reducing ROS, which was evident from decreased immunostaining for nitrotyrosine in the tibia section [38]. This evidence prompted us to examine whether delphinidin stimulates OB differentiation in intact MC3T3-E1 cells, and attenuates NF-κB activation through protecting I-κB from protein nitration.

Here, we investigated the effects of delphinidin-enriched MBE on bone metabolism both in vitro, especially its effects on OB differentiation in relation to protein nitration of I-κB, and in two osteopenia mouse models. In one, bone loss was due to estrogen deficiency, and the other was a hormone-independent model with the aim to cover several possibilities of bone loss that may occur in humans, to examine its efficiency in protecting against bone loss over time by daily oral administration.

## 2. Materials and Methods

### 2.1. Maqui Berry Extract

Delphinol^®^ and maquisupreme^®^ are the brand names of a standardized maqui berry extract, more precisely a highly water-soluble powder extract produced under GMP (Good Manufacturing Practice) conditions by Anklam Extrakt GmbH, Anklam, Germany. Delphinol^®^ is standardized to contain a minimum of 25% delphinidins and a minimum of 35% total anthocyanins, resulting in an exceptionally high antioxidant capacity assayed by oxygen radical absorbance capacity (ORAC) test system. The method provides the total antioxidant potency of a nutrition/food product against five reactive oxygen species found in the body (peroxyl radicals, hydroxyl radicals, peroxynitrite, super oxide anion, and singlet oxygen). The ORAC of maqui berry extract was determined by Brunswick Laboratories (Southborough, MA, USA), which certified (issued on 11/01/2013) a total ORAC of 30,852 μmole trolox equivalents per one gram of extract. Trolox [6-hydroxy-2,5,7,8-tetramethylchroman-2-carboxylic acid], a water-soluble vitamin E analog that is utilized as a positive control and calibration standard marker, and ORAC analytical results are expressed as trolox equivalents. The methodological details of this assay are described elsewhere [39].

### 2.2. Effects of MBE on the Differentiation of MC3T3-E1 Cells

MC3T3-E1 cells, a preosteoblastic mouse cell line, were maintained and differentiated as described previously [40]. A mineralized matrix stained with Alizarin Red-S was photographed, and the intensity was measured using Molecular Imager (Bio-Rad, Hercules, CA, USA). For alkaline phosphatase (ALP) activity measurement, cells were sonicated in 0.1 M Tris-HCl (pH 7.2, 0 °C) containing 0.1% Triton X-100 and examined using a LabAssay ALP kit (Wako Pure Chemical Industries Ltd., Osaka, Japan) based on the Bessey–Lowry method. Quantitative real-time PCR (qPCR) was performed in a Thermal Cycler Dice Real Time System (TP-870; Takara, Tokyo, Japan) using SYBR Premix Ex Taq II (Takara Bio Inc., Shiga, Japan) according to the manufacturer’s protocol. The sequence of the specific primers for the qPCR is listed in Table 1. The level of mRNA expression was normalized with that of Actb (β-actin) expression.

### 2.3. NF-κB Nuclear Translocation and Tyrosine Nitration of I-κB

MC3T3-E1 cells were grown for 3 days in the absence or presence of 25.0 μg/mL of MBE; then, they were incubated with 10 μg/mL of lipopolysaccharide (LPS, *Escherichia coli* O111:B4, Merck KGaA, Darmstadt, Germany) for 3 h. For the immunohistochemical examination, cultures were stained with goat anti-NF-κB/p65 antibody (C-20, sc-372-G, Santa Cruz Biotechnology, Inc., Dallas, TX, USA), rabbit anti-I-κB antibody (E130, ab32518, Abcam, Cambridge, UK), and mouse anti-nitrotyrosine antibody (7A12AF6, ab110282; recognize only protein-bound nitrotyrosine, Abcam) followed by appropriate Alexa Fluor 488^®^ or 594^®^-labeled secondary antibodies (Invitrogen Co., Carlsbad, CA, USA). After immunostaining, cells were observed using a laser scanning confocal microscope (FV1000; Olympus Optical Co., Tokyo, Japan). For the immunoprecipitation, samples (1 × 10^7^ cells each) were lysed with lysis buffer (25 mM Tris·HCl, pH 7.5, 150 mM NaCl, 0.1% Triton X-100, 2mM EDTA, 1 mM PMSF) containing a protease inhibitor cocktail (Merck KGaA). I-κB-α was immunoprecipitated with mouse monoclonal anti-I-κB-α antibody (H-4, sc-1643, Santa Cruz Biotechnology, Inc.) overnight at 4 °C with continuous rotation and purified using protein G magnetic beads (“SureBeads^TM^ Protein G beads”, Bio-Rad). The immunocomplex was separated by 15% sodium dodecyl sulfate polyamide gel electrophoresis, transferred onto Immobilon-P, and then incubated with mouse monoclonal anti-nitrotyrosine antibody (39B6, sc-32757, Santa Cruz Biotechnology, Inc.), followed by biotin-conjugated rabbit polyclonal anti-mouse antibody (Jackson ImmunoResearch, West Grove, PA, USA) and avidin-conjugated horseradish peroxidase (Bio-Rad). Signals were detected by chemiluminescence reagent (Luminata^TM^ Forte Western HRP substrate, Merck KGaA).

### 2.4. OC Differentiation

Whole bone marrow cells were obtained from the femora and tibiae of C57BL/6J mice (10-week-old, male) according to the protocol approved by the Animal Experimental Committees of Showa University (code number: 17050, 04/01/2017). Cells (1 × 10^5^ cells per cm^2^) were incubated in α-MEM (Gibco) containing 10% FBS supplemented with 4000 U/mL Leukoprol (M-CSF: macrophage colony stimulating factor, JCR Pharmaceuticals Co. Ltd., Hyogo, Japan) for 2 days to induce bone marrow macrophages (BMM). BMM were further incubated with 50 ng/mL of soluble receptor activator of NF-κB ligand (sRANKL, Wako Pure Chemical Industries Ltd.) in the presence of Leukoprol (4000 U/mL) for 3 days to differentiate into maturated OC. BMM were incubated in the presence of 0–111 μg/mL MBE during only the first 2 days with Leukoprol, then for 3 days with Leukoprol and sRANKL, or throughout the culture period. The culture medium was changed every second day. OC differentiation was examined by measuring the intensity of tartrate-resistant acid phosphatase (TRAP) staining at 540 nm using a microplate reader (Infinite 200, Tecan Japan Co. Ltd., Kawasaki, Japan). qPCR was performed with a StepOne Real-Time PCR system (Thermo Fisher Scientific, Waltham, MA, USA) using SYBR Green (Toyobo Co. Ltd., Osaka, Japan). The sequence of the specific primers for the qPCR is listed in Table 1. The level of mRNA expression was normalized with that of glyceraldehyde-3-phosphate dehydrogenase (*Gapdh*) expression. Cell proliferation of BMM was examined by 5-bromo-2′-deoxyuridine (BrdU) incorporation assay. Briefly, bone marrow cells (3 × 10^5^ cells per cm^2^) were cultured in α-MEM containing 10% FBS supplemented with 4000 U/mL Leukoprol for 2 days and incubated for 4 h in the presence of BrdU. BrdU incorporation was detected based on a colorimetric ELISA assay kit (Cell Proliferation ELISA, BrdU; Roche, Basal, Switzerland).

### 2.5. Pit Formation

Bone marrow cells (2 × 10^7^ cells) and stromal/osteoblastic cells (UAMS-32, 1.25 × 10^6^ cells) were co-cultured in α-MEM containing 10% inactivated FBS (Gibco) in the presence of 10^−8^ M 1α,25-dihydroxyvitamin D_3_ (1α,25(OH)_2_D_3_; Sigma-Aldrich, St Louis, MO, USA) and 10^−6^ M prostaglandin E_2_ (Sigma-Aldrich) on culture dishes precoated with 5 mL of collagen gel matrix (3 mg/mL; Nitta Gelatin Inc., Osaka, Japan) for 8 days. The medium was changed every 4 days. OC were recovered by a mixture of 0.4% collagenase (Wako Pure Chemical Industries Ltd.) and 0.2% dispase protease (Wako Pure Chemical Industries Ltd.). OC obtained from the above-mentioned co-culture were transferred into either (i) 24-well culture plates, (ii) dentine slices (6 mm in diameter; MS Labo System, Yokohama, Japan) in 24-well culture plates, or (iii) a calcium phosphate-coated plate in 24-well plates, and incubated in the presence of MBE (0–333 μg/mL) for a further 24 h. Cultures were evaluated as follows: (i) fixed OC were stained for TRAP activity using Naphtol AS-MX phosphate (3-hydroxy-2-naphtho-2′,4′-xylidide phosphate, Sigma-Aldrich) and Fast red (Sigma-Aldrich) dissolved in 0.1 M acetic buffer (pH 5.0) containing 1% tartaric acid. The area of TRAP staining was measured under a microscope (BZ-X700, Keyence Co., Osaka, Japan); (ii) dentine slices were cleaned by ultrasonication (Sonics and Materials Inc., Newtown, CT, USA) to remove adherent cells in 0.5 M NH_4_OH (Wako pure chemical industries Ltd.), and stained with toluidine blue (Merck KGaA) to visualize resorption pits. The area of pits on slices was measured using a microscope (BZ-X700), and (iii) calcium phosphate-coated plates were cleaned with 5% sodium hypochlorite for five minutes to remove adherent cells. The resorbed area of plates was measured using a microscope (BZ-X800; Keyence Co., Osaka, Japan).

### 2.6. In Vivo Examination in sRANKL and Ovariectomy-Induced Osteopenic Mouse Models

To examine the effect of MBE on bone loss in vivo, we used an sRANKL-induced osteopenic mouse model [41]. C57BL/6J mice (7-week old, female, *n* = 30) were obtained from CLEA Japan (Tokyo, Japan). Mice were randomly assigned to five groups: controls (*n* = 6), sRANKL-induced osteopenia (vehicle, *n* = 6), low-dose MBE (0.25 mg/mouse/day, which equals 12.5 mg/kg/day, n = 6), intermediate-dose MBE (0.5 mg/mouse/day, which equals 25.0 mg/kg/day, *n* = 6) or high-dose MBE- (0.75 mg/mouse/day, which equals 25.0 mg/kg/day, *n* = 6). The mice were administered with sRANKL (2.0 mg/kg, intraperitoneal; Oriental Yeast Co., Ltd., Kyoto, Japan) two times at an interval of 24 h. For 18 mice, the daily MBE ingestion using a flexible polyethylene tube fitted with a blunt end stainless tip (0.2 mL/mouse) started 7 days prior to the administration of sRANKL, and continued for 14 days. MBE dosages were decided with reference to the daily supplement dosage for humans; i.e., 60 mg for humans of 60 kg, which is thought to be equivalent to 0.25 mg/mouse/day [42]. The remaining 12 mice were given the same volume of water using the stomach tube. We further evaluated the effect of MBE using ovariectomized (OVX) mice. C57BL/6J mice (9-week-old, female) that underwent either sham operation (*n* = 6) or OVX (*n* = 32) one week before were purchased from CLEA Japan. OVX mice were randomly assigned into four groups (*n* = 8): OVX-control (vehicle, *n* = 8), low-dose MBE (0.25 mg/mouse/day, *n* = 8), intermediate-dose MBE (0.5 mg/mouse/day, *n* = 8), and high-dose MBE (0.75 mg/mouse/day, *n* = 8) groups. A week after OVX surgery, the indicated dosage of MBE was ingested, as mentioned above, daily for 28 days. Sham-operated and OVX-control mice were given the same volume of water. Mice were housed in an air-conditioned room (temp, 22 ± 2 °C; humidity, 50%; light/dark cycle, 12 h) with free access to food and water. At the end of the experiment, the mice were anesthetized with a mixture of medetomidine hydrochloride, midazolam, and butorphanol tartrate. Plasma was collected in a heparinized syringe by cardiac puncture and stored at –35 °C until the malondialdehyde (MDA) measurement, which was performed using OxiSelect^TM^ TBARS Assay kit (Cell Biolabs Inc., San Diego, CA, USA). Femurs harvested at the end of treatment were fixed with 10% neutral formalin overnight. After rinsing with tap water, bones were immersed in 70% ethanol and stored at 4°C until analyses. Animal experiments were performed in compliance with the commonly accepted ‘3Rs‘—Replacement, Reduction, Refinement—according to the protocol, which was approved by the “Experimental Animal Center in Ohu University” (code number: 2017-7, 01/04/2017 and 2018-9, 01/04/2018).

### 2.7. Bone Morphometric Analyses

The bone morphometric parameters and microarchitectural properties of the distal femurs were examined using a microcomputed tomography (micro-CT) system (ScanXmate-L090H; Comscantecno Co., Yokohama, Japan), as described previously [30]. For quantitative analysis of bone structural indices, BV/TV (ratio of bone volume to tissue volume), Tb.Th (trabecular thickness), Tb.N (trabecular number), Tb.Sp (trabecular separation), and Nd/Tm (ratio of nodes to termini) were determined using TRI/3D-BON software (RATOC System Engineering Co., Tokyo, Japan) according to the guidelines for the assessment of bone microstructure in rodents using microcomputed tomography [43]. For the evaluation of dynamic parameters, mice were injected subcutaneously with tetracycline hydrochloride (20 mg/kg) and calcein (10 mg/kg), both Ca-chelating fluorescent molecules, 5 days and 2 days prior to sacrifice, respectively. Femora were stained with Villanueva-Goldner bone stain (Wako Pure Chemical Industries Ltd.) and then embedded in methyl methacrylate (MMA) without decalcification. Coronal sections of the distal femurs were observed under a conventional fluorescent microscope (model 200; Carl Zeiss, Oberkochen, Germany). The data are expressed according to the guidelines of the ASBMR Histomorphometry Nomenclature Committee [44].

### 2.8. Statistics

Statistics were analyzed using one-way analysis of variance for comparison among all groups. The Tukey test was used for post hoc pair-wise comparisons. Statistical analyses were performed using KaleidaGraph (HULINKS Inc., Tokyo, Japan) or JMP Pro 13 software (SAS Institute Inc., Cary, NC, USA). All values are expressed as mean ± standard deviation (SD) unless otherwise indicated. A *p*-value less than 0.05 was considered statistically significant (*p < 0.05, **p < 0.01, ***p < 0.001).

## 3. Results

### 3.1. Effects of MBE on OB Differentiation

**Figure 1:**
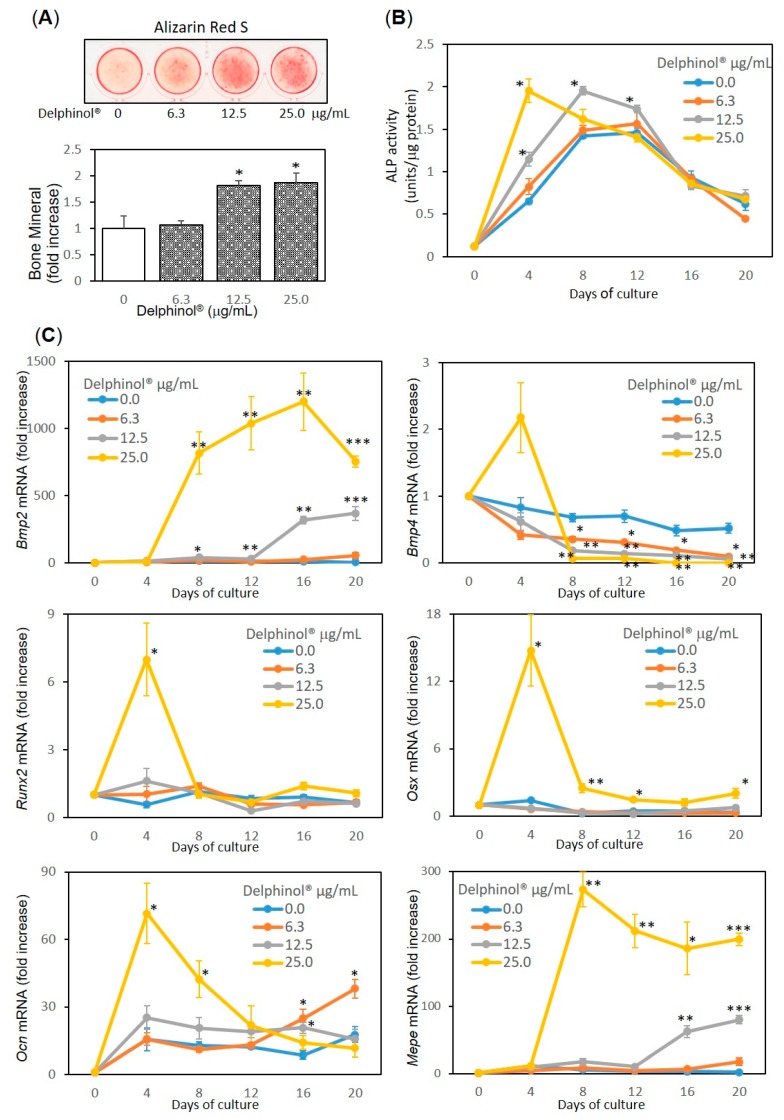
Stimulatory effects of Delphinol^®^ on osteoblastogenesis. (**A**) Mineralization of MC3T3-E1 cells. Confluent culture was incubated with Delphinol^®^ at indicated concentrations for 16 days. At the end of incubation, cells were washed then fixed with 70% ethanol, and stained with Alizarin red S. (**B**) Alkaline phosphatase (ALP) activity of MC3T3-E1 cells. Confluent culture was treated with Delphinol^®^. At the end of incubation,. ALP activity was measured in the cell lysate. (**C**) Expression of osteoblast (OB) differentiation-related genes in MC3T3-E1 cells. Confluent culture was treated with or without Delphinol^®^. At the end of incubation, total RNA was extracted, reverse-transcribed, and used for qPCR. Values are expressed as mean ± SEM (*n* = 3–4). *p < 0.05, **p < 0.01, ***p < 0.001.

MBE (12.5 and 25.0 μg/mL) stimulated the multilayered proliferation of MC3T3-E1 cells, resulting in a significant increase in mineral deposition on day 16, as shown in red with Alizarin red S staining (Figure 1A). In the course of bone nodule formation, MBE (12.5 and 25.0 μg/mL) significantly stimulated ALP activity, which is an OB marker (Figure 1B).

qPCR analysis of MC3T3-E1 cell cultures (Figure 1C) displayed that bone morphogenetic protein (Bmp) 2 mRNA was upregulated 400-fold and 1200-fold by 12.5 and 25.0 μg/mL of MBE treatment on day 16, respectively. *Bmp4* mRNA was upregulated twofold on day 4 by 25.0 μg/mL of MBE, although this was not significant (*p* = 0.11). In contrast, *Bmp4* mRNA was downregulated significantly by 12.5 μg/mL throughout the culture period, and on and after day 8, it was downregulated significantly by 25.0 μg/mL of MBE. The mRNA expression of runt-related transcription factor 2 (*Runx2*) and osterix (*Osx*), which are both bone-specific transcription factors known to regulate bone homeostasis, were upregulated by 25.0 μg/mL of MBE. The expression of matrix extracellular phosphoglycoprotein (*Mepe*), which is an extracellular matrix in bone, and osteocalcin (*Ocn*), a marker for bone formation, were upregulated by 25.0 μg/mL of MBE, and to a lesser extent and at a later time point, by the addition of 12.5 μg/mL of MBE.

### 3.2. Inhibitory Effects of MBE on NF-κB Nuclear Translocation and Nitration of I-κB

**Figure 2:**
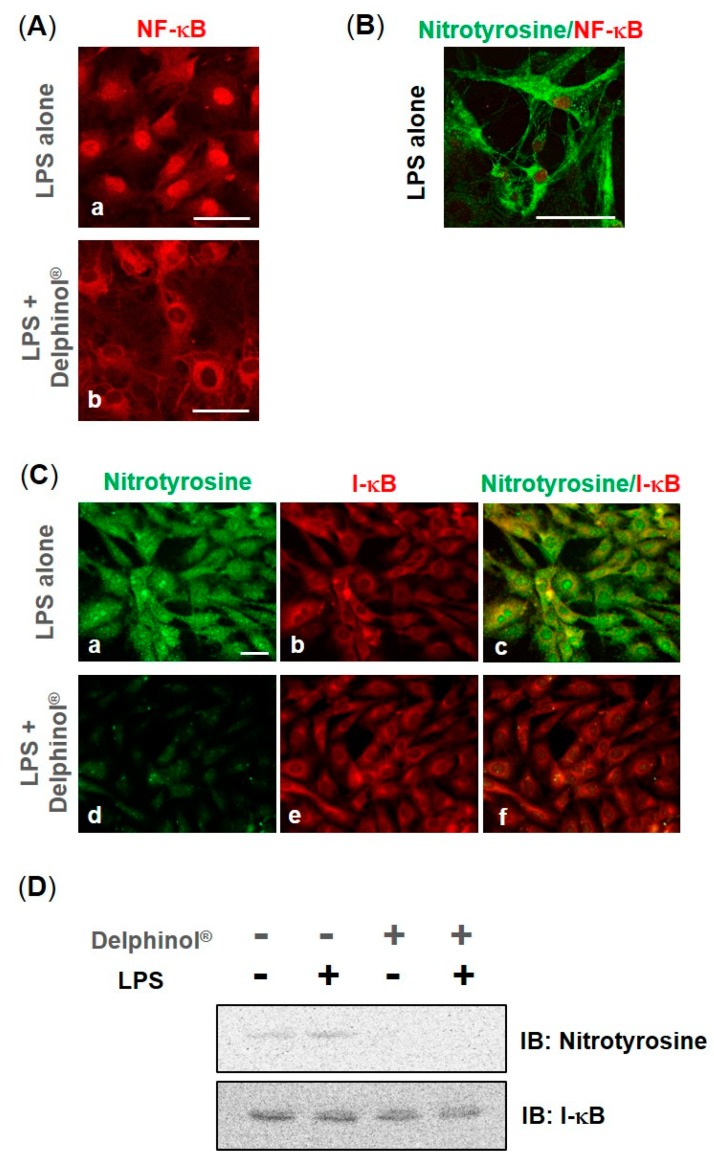
Effects of Delphinol^®^ on the transnucleation of NF-κB and tyrosine nitration of I-κB. (**A**–**C**) Immunofluorescent staining of MC3T3-E1 cells for NF-κB/p65, nitrotyrosine, and I-κB. Cells were grown for 3 days in the absence or presence of 25.0 μg/mL Delphinol^®^, then incubated with 10 μg/mL LPS for 3 h. Scale bars = 50 μm. (**D**) Cell samples were immunoprecipitated with anti-I-κB antibody, and then blotted with either anti-nitrotyrosine antibody or anti-I-κB antibody.

Immunostaining examination for NF-κB/p65, an active subunit of NF-κB, revealed that 25.0 μg/mL MBE almost completely inhibited the LPS-induced transnucleation of p65, which is clearly recognized as the cytoplasmic staining (Figure 2(Ab)). In addition, nitrated protein tyrosine was detected in the cytoplasm in LPS-treated cells concomitant with the NF-κB transnucleation (Figure 2B). The partial colocalization of nitrotyrosine and I-κB (Figure 2(Cc,f)) indicated that I-κB was one of the proteins that was nitrated by LPS-induced oxidation. The analysis of the immunoprecipitation assay demonstrated that I-κB was nitrated on tyrosine by LPS stimulation, and MBE almost fully suppressed this reaction (Figure 2D), supporting the colocalization of nitrotyrosine and I-κB in the immunostaining.

### 3.3. Inhibitory Effects of MBE on OC Differentiation

**Figure 3:**
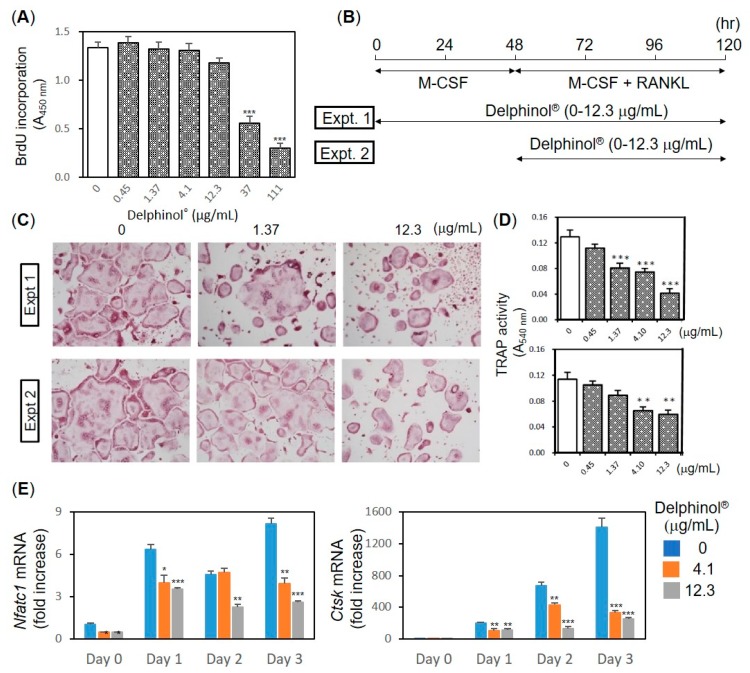
Effects of Delphinol^®^ on in vitro osteoclastogenesis. (**A**) Cell proliferation of BMM was examined by BrdU incorporation assay. Bone marrow cells (3 × 10^5^ cells per cm^2^) were cultured in α-MEM containing 10% FBS supplemented with 4000 U of Leukoprol for 2 days and incubated for 4 h in the presence of BrdU. BrdU incorporation was detected based on a colorimetric ELISA assay kit. (**B**) Experimental protocol for osteoclastogenesis. (**C**) Representative photographs of tartrate-resistant acid phosphatase (TRAP) staining in osteoclasts (OC) cultures. Scale bars = 50 μm. (**D**) Anti-osteoclastogenic activity of maqui berry extract (MBE) was examined by the intensity of TRAP staining at 540 nm on a spectrophotometer. (**E**) qPCR analyses of *Nfatc1* and *CtsK* mRNA expression. The level of mRNA expression was normalized with that of *Gapdh.* Values are expressed as mean ± SD (*n* = 3–4). *p < 0.05, **p < 0.01, ***p < 0.001.

Next, we investigated the effects of MBE on osteoclastogenesis by using primary mouse bone marrow cells. Our preliminary experiment, in which MBE (0–111 μg/mL) was added only during the first two days of culture before being changed to fresh media containing Leukoprol and RANKL, showed that a higher dose of MBE had cytotoxic effects on OC precursors (data not shown). To determine the MBE concentration that was expected to impair the precursor proliferation, the effects of MBE on BrdU incorporation activity were examined (Figure 3A). As shown in Figure 3A, MBE higher than 37.0 μg/mL exhibited a cytotoxic effect on bone marrow-derived monocyte/macrophage precursor cells (BMM). Therefore, we used MBE lower than 12.3 μg/mL in the osteoclastogenesis assay. MBE was present throughout the 120-h culture period in Experiment 1, whereas MBE was present only during the differentiation of BMM into mature multinucleated OC by RANKL in Experiment 2 (Figure 3B). Dose-dependent inhibitory effects of MBE at concentrations higher than 1.37 μg/mL were observed in both experimental protocols, which indicated that MBE dose-dependently inhibited the maturation of OC (Figure 3C,D). qPCR analysis of OC showed that MBE inhibited the RANKL-induced upregulation of mRNA of *Nfatc1*, which is a master transcriptional factor for osteoclastogenesis [45], and *CtsK*, which is a cysteine protease predominantly expressed in OC, at concentrations higher than 4.1 µg/mL dose-dependently (Figure 3E).

### 3.4. Effects of MBE on Pit Formation

**Figure 4:**
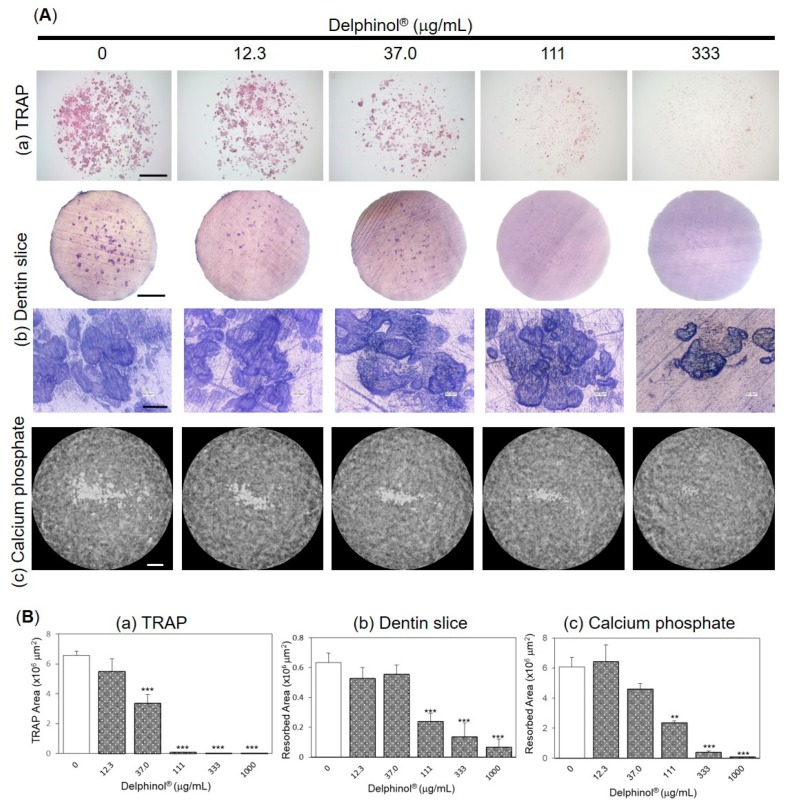
Effects of Delphinol^®^ on pit formation by mature OC. (**A**) Representative photographs of (**a**) TRAP staining, (**b**) resorption pit on dentine slices, and (**c**) resorption of calcium phosphate in OC cultures. (**B**) Anti-resorption activity of MBE was evaluated by measuring the area per well. Values are expressed as mean ± SD (*n* = 3–4). **p < 0.01, ***p < 0.001. Scale bars are 2 mm except for the lower panel of Figure 4(Ab) (20 μm).

In another experimental approach, we examined the inhibitory effects of MBE on the function of mature OC generated from the co-culture of bone marrow cells and stromal/osteoblastic UAMS-32 cells. MBE significantly inhibited the further maturation of OC transferred onto a plastic plate at concentrations higher than 37.0 μg/mL (Figure 4(Aa,Ba)), and the resorption of both dentin slices (Figure 4(Ab,Bb)) and calcium phosphate substrate (Figure 4(Ac,Bc)) at concentrations higher than 111 μg/mL. Resorption pits created by OC in the presence of MBE were normal in size, whereas the numbers of pits decreased dose-dependently (Figure 4(Ab)).

### 3.5. Effects of Administration of MBE on Bone Loss in Osteopenic Mice

**Figure 5:**
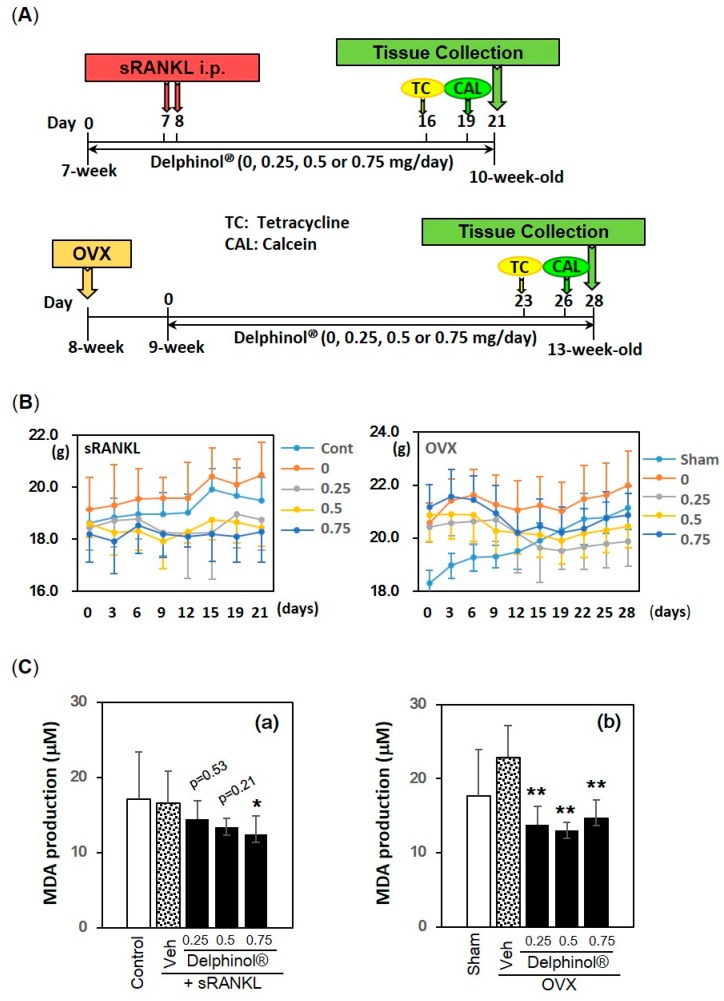
In vivo experimental protocol and effects of Delphinol^®^ on body weight and plasma malondialdehyde (MDA) level. (**A**) Experimental protocol of sRANKL-induced (upper panel) and ovariectomized (OVX) (lower panel)-induced osteopenic mouse models. All mice were injected with two fluorescent molecules, tetracycline and calcein, sequentially prior to sacrifice for the purpose of kinetic analyses in bone histomorphometry. (**B**) Changes in body weight during the experiment. (**C**) Plasma MDA levels in sRANKL-induced (**a**) and OVX (**b**)-induced osteopenic mice. Values are expressed as mean ± SD (*n* = 5–8). *p* values and significance levels are the comparisons between vehicle-treated and MBE-treated groups. *p < 0.05, **p < 0.01.

To clarify the relevance of our in vitro findings of MBE on biological function in vivo, we examined the potential anti-osteopenia effect of MBE on two osteopenic mouse models. One model mimics estrogen-deficiency in humans (OVX), and the other was a rapid (<50 h) bone loss model, based on strong stimulation with sRANKL, that was reported to be indistinguishable from that in the OVX model [41]. The average body weights in the sRANKL-induced osteopenia mice (sRANKL) were 18.6 g (16.0–21.0 g) and 19.1 g (16.7–22.3 g), at the start and the end of the experiment, respectively. The sham-operated mice (sham) were 19.0 g (17.6–20.3 g) and 21.2 g (20.0–21.9 g); ovariectomized (OVX) mice were 19.6 g (17.9–21.4 g) and 20.8 g (18.0–23.8 g). All the mice were healthy with regard to food and water intake. None exhibited changes in behavior, activity, and general appearance, and changes in body weight remained within normal ranges: a 0.5% increase in OVX mice to a 15.4% increase in sham-operated mice (Figure 5B). There were no abnormalities in the organs that were harvested at the end of the trials, and organ weight was within normal ranges, varying according to mice body size. Moreover, MBE treatment reduced the body weight gain and the abdominal fat accumulation in OVX mice. In order to examine the antioxidative activity of MBE in vivo, the levels of MDA, a marker for oxidative stress, were measured in the plasma samples collected at the end of experiments. As shown in Figure 5C, the oral administration of MBE (0.25, 0.5 and 0.75 mg/day) significantly decreased the MDA levels in OVX-induced osteopenic mice (Figure 5(Cb)), whereas only the highest dose of MBE (0.75 mg/day) exhibited the statistical significant inhibitory effect in sRANKL-injected mice (Figure 5(Ca)).

**Figure 6:**
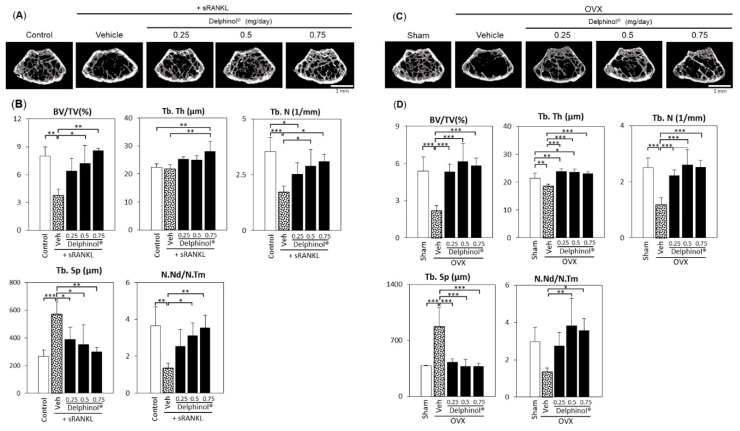
Protective effect of Delphinol^®^ on bone loss in sRANKL and OVX-induced osteopenia mice. (**A**) Representative microcomputed tomography (micro-CT) images of the distal femurs of intact mouse (control), sRANKL-induced osteopenia mouse (vehicle), and MBE-treated sRANKL-induced osteopenia mice (MBE; 0.25, 0.5, 0.75 mg/mouse/day). (**B**) Microarchitectural indices of second trabecular spongiosa of the distal femurs as measured by micro-CT. (**C**) Representative micro-CT images of the distal femurs of sham-operated mouse (Sham), OVX-induced osteopenia mice (vehicle), and MBE-treated OVX-induced osteopenia mice (MBE; 0.25, 0.5, 0.75 mg/mouse/day). (**D**) Microarchitectural indices of second trabecular spongiosa of the distal femurs as measured by micro-CT. Values are expressed as mean ± SD (*n* = 5–8). *p < 0.05, **p < 0.01, ***p < 0.001.

As depicted in the images generated from micro-CT scans of the vertical midsection of metaphyseal femurs of sRANKL-induced (Figure 6A), and OVX-induced (Figure 6C) osteopenia, the area vacant of trabecular bone (seen in the vehicle group) decreased in size and filled with trabecular bone in the MBE-ingested group. Changes in the bone morphometric parameters that were analyzed using micro-CT demonstrated that MBE significantly increased BV/TV, Tb.Th, and Tb.N, and decreased Tb.Sp, indicating a gain in trabecular bone mass; it also increased N.Nd/N.Tm, reflecting the improved spatial connectivity of trabecular networks (Figure 6B,D).

**Figure 7:**
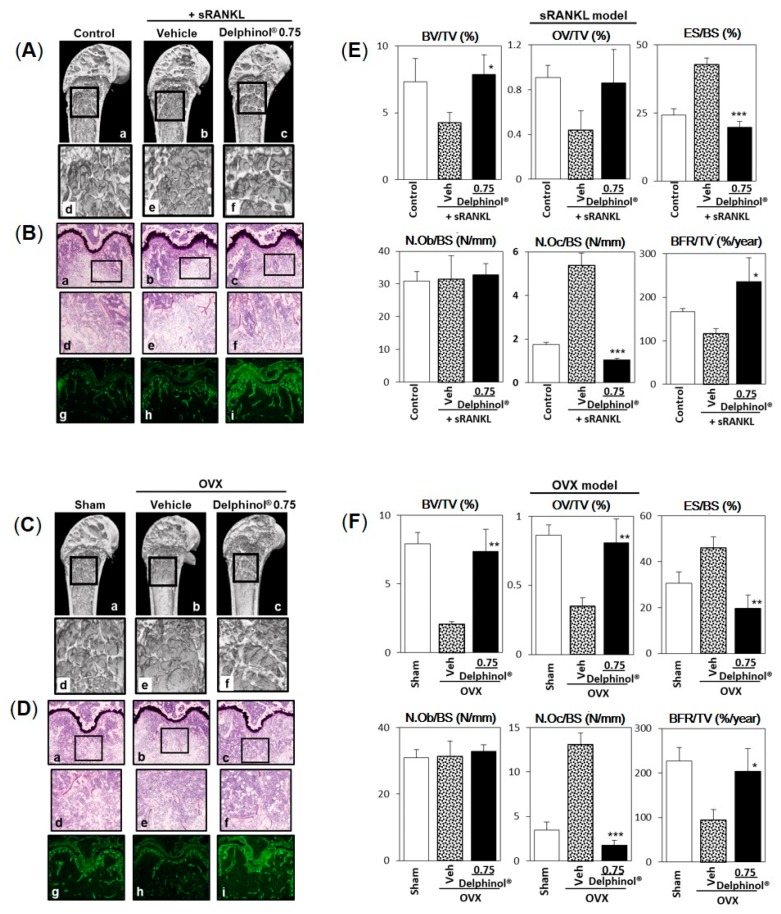
Histomorphometrical analyses of effect of Delphinol^®^ on bone loss in sRANKL-induced and OVX-induced osteopenic mouse models. (**A**) Representative three-dimensional micro-CT images of the sagittal midsection of distal end of femurs in intact mice (Figure 7(Aa,Ad), control), sRANKL-induced osteopenic mice (Figure 7(Ab,Ae), vehicle), and Delphinol^®^ 0.75 (mg/mouse/day)-administered sRANKL-induced osteopenic mice (Figure 7(Ac,7Af)). (**C**) Representative micro-CT images of the sagittal midsection of distal femurs in sham-operated mouse (Sham), OVX-induced osteopenic mouse (vehicle), and a Delphinol^®^ 0.75 (mg/mouse/day)-treated OVX-induced osteopenic mouse. (**B**,**D**) Representative photographs of distal femur sections stained by Villanueva staining in sRANKL- and OVX-induced osteopenic mice, respectively. Figures in the middle panel correspond to the squares in the upper panel. Figures in the lowest panel are fluorescent (calcein) images of the same field of Villanueva staining. (**E**,**F**) Histomorphometric parameters were determined by morphometric analyses of fluorescent (tetracycline and calcein)-labeled femurs according to the methods of the ASBMR Histomorphometry Nomenclature Committee [44]. Values are expressed as mean ± SD (*n* = 5).; Significance was tested between vehicle and 0.75 mg/mouse/day Delphinol^®^. *p < 0.05, **p < 0.01, ***p < 0.001.

To clarify whether the increase in trabecular bone volume by MBE treatment resulted from only inhibited bone resorption or a combination with stimulated bone formation, further investigation was conducted in the highest MBE (0.75 mg/mouse/day) groups compared to the vehicle groups. The three-dimensional sagittal midsection of distal femurs constructed from micro-CT scans revealed that trabecular bone, as observed in MBE groups of both osteopenia models, sRANKL, and OVX (Figure 7(Ac,f) and Figure 7(Cc,f)) was thicker compared with that of the control or sham group mice, which may indicate that MBE not only inhibited bone resorption, but also stimulated bone formation, which was consistent with the in vitro findings (Figure 1). Also, a stronger fluorescence of calcein injected 2 days prior to the sacrifice in Villanueva-stained primary spongiosa of MBE groups (Figure 7(Bi,Di)) indicated that new mineralization was induced by MBE administration. Bone histomorphometry analyses showed that MBE significantly increased BV/TV, OV/TV, and BFR/TV (Figure 7E,F), and significantly decreased ES/BS and N.Oc/BS (Figure 7E,F) in both the sRANKL-induced and OVX-induced osteopenic mouse models.

## 4. Discussion

The bioavailability of anthocyanins had been reported to be extremely low due to very limited circulating concentrations in blood, especially detected as glycosylated forms or aglycones, in the past. However, recently, it has been reported that anthocyanins and their metabolites are found in the gastrointestinal tract, as well as invarious organs and tissues by undergoing extensive enterohepatic circulation [46]. A large cross-sectional study investigated the association between habitual flavonoid consumption and BMD (bone mineral density) in 3160 women. The study concluded that the consumption of flavonoids, most importantly, anthocyanins (median consumption: 13.7 mg/day), had bone protective effects [47]. Karksen et al. demonstrated that supplemental capsules containing purified anthocyanins isolated from bilberries and black currant reduced plasma levels of proinflammatory cytokines and chemokines in healthy adults [28]. In addition, Goszcz et al. showed that delphinidin and gallic acid, a major metabolite in the body, protected against oxidative stress through a mechanism that was associated with increased glutathione in cultured endothelial cells [48]. Anthocyanin content differs quantitatively and qualitatively in distinct foods and supplemental mixtures. In general, extracted anthocyanin compounds consist of several types of anthocyanins derived from distinct aglycones which include delphinidin, cyanidin, and peonidin. These are the major anthocyanidins found in berry fruits [49]. To be more specific, we used purified delphinidin in our previous study, which had shown that aglycone itself was a potent inhibitor of osteoclastogenesis in RAW 264.7 cells, and exerted bone protective effects in osteoporotic mice [30]. In the present study, we used MBE powder (Delphinol^®^), prepared from fresh maqui berries by water extraction followed by purification on resin and standardized to contain >25% delphinidins and >35% total anthocyanins. A recent study using MBE powder showed that after a single-dose supplementation in humans, both native and metabolites of anthocyanins were found in their plasma, possibly indicating a higher bioavailability of MBE [50]. The present study using bone marrow precursor cells showed that MBE inhibited osteoclastogenesis dose-dependently at concentrations lower than cell toxicity through downregulating *Nfatc1* and *CtsK* mRNA, which are known to be downstream genes of NF-κB. Furthermore, MBE treatment also suppressed TRAP-positive mature OC formation in the co-culture of bone marrow cells and UAMS-32 cells, as well as its subsequent activation, which was measured by pit assay. A few studies have reported that flavonoids could be inhibitors of the RANK/RANKL signaling-related molecules, such as NF-κB and *NFATc1* [51,52], thus supporting the findings of both our previous study [30] and the present study.

In addition to inhibiting osteoclastogenesis, MBE stimulated the OB differentiation of MC3T3-E1 cells, as indicated by enhanced mineralized nodule formation and ALP activity, through upregulating *Bmp2*, *Runx2*, *Osx*, *Ocn*, and *Mepe* mRNA. To elucidate if MBE exerts the stimulatory effects on OB through antioxidative activity, we investigated the response to exogenous LPS, a bacterial component, in MC3T3-E1 cells. It is well recognized that host cells, including neutrophils and macrophages, produce large amounts of ROS and reactive nitrogen species (RNS) to kill bacteria and protect themselves against invading pathogens, which results in serious tissue damage and cell death through NF-κB activation. As expected, MBE almost completely inhibited the LPS-induced nuclear translocation of NF-κB. Therefore, the inhibitory effect of MBE on LPS-induced NF-κB activation is likely the underlying mechanism of MBE-induced stimulation of OB differentiation, as supported by the observations showing that NF-κB activation suppressed osteoblastogenesis [31,32,33]. Furthermore, the present study suggests that the attenuation of LPS-induced tyrosine nitration of I-κB and resulting suppression of NF-κB nuclear translocation is a possible mechanism for OB activation. That the MBE powder we used was proved to possess the antioxidant activity against superoxide anion and peroxynitrite supports our hypothesis that MBE may scavenge superoxide and/or peroxynitrite to inhibit the tyrosine nitration of I-κB, which can cause NF-κB activation for longer period of time than the phosphorylation of I-κB [38,53]. However, since crosstalk between Wnt/β-catenin, which is known to play a critical role in osteogenic differentiation, and the NF-κB signaling pathway has been reported in inflammation [54], further investigation is necessary to clarify the mechanisms of MBE action on bone metabolism both in vitro and in vivo.

Animal experiments performed to determine the physiological relevance of the above-mentioned in vitro findings clearly demonstrated that MBE had the ability to protect against and/or reverse bone loss in both sRANKL-induced and OVX-induced osteopenic mice. These results showed that the administration of MBE enabled balancing bone metabolism, resulting in adequate bone remodeling. Furthermore, the fluorescence of calcein in the osteoid and primary spongiosa was increased by MBE, indicating that bone formation was stimulated in both sRANKL-induced and ovariectomy-induced osteopenic mice by MBE administration.

A decrease in BMD is shown to be correlated with a higher oxidative stress index and oxidative status in the plasma of postmenopausal osteoporotic patients [55]. The production of ROS/RNS during physiological metabolism is an integral feature of aerobic organisms. Endogenous enzymatic and non-enzymatic antioxidants counteract the toxic effects of this oxidative/nitrosative stress to balance the redox state. However, the excessive production of ROS/RNS due to an imbalance between the formation and removal of reactive molecules by antioxidants may cause damage in various tissues. Therefore, we examined the plasma levels of MDA, a product of polyunsaturated fatty acid peroxidation and a marker for oxidative stress, to elucidate the possible involvement of antioxidant activity of MBE in the protection of bone loss. As expected, the plasma MDA levels that increased in OVX mice (*p* = 0.18 versus sham-operated mice) were significantly decreased by oral administration with MBE (0.25–0.75 mg/mouse/day) for four weeks, indicating a possible role of antioxidant activity of MBE in bone protection. In sRANKL-induced osteopenic mice, only the highest dosage (0.75 mg/mouse/day) was able to induce a significant decrease in the plasma MDA. The shorter period of MBE administration in sRANKL model compared with OVX model, i.e., two weeks compared with four weeks, could be the reason for the weaker antioxidative effects. Furthermore, in order to confirm that the plasma MDA level would not be increased with sRANKL injection, MDA should be measured in the plasma samples collected at an earlier time point after sRANKL injection. Considering ROS/RNS can be produced and localized ubiquitously both in intracellular and extracellular spaces because they freely diffuse through the cell membranes, the possibility that MBE acts indirectly via eliminating ROS/RNS even outside the target cells, otherwise causing activation of NF-κB, is raised. We hypothesize that this pathway may be a mechanism through which MBE simultaneously impacts on both types of bone cells, OC and OB, and thereby improves bone formation/resorption. As it has been well recognized that there is a coupling between OB and OC cells to maintain physiological bone metabolism in adults, the above-mentioned bidirectional functions of antioxidant MBE may provide an advantage to protect bone health. Supporting this hypothesis, the literature showed that osteoclastogenesis was stimulated by ROS-induced NF-κB activation, whereas OB differentiation [11,12] and bone formation in vivo [31,32] were inhibited by ROS-induced NF-κB activation.

The higher the peak bone mass, the stronger bones will be in later life. Given that a dietary approach is essential for bone health [11], and since osteoporosis is now thought to be a lifestyle-related disease, the ideal regimen for a nutritional supplement may be a low dosage supplement, but for a longer duration and commencing from a younger age. Anthocyanins have been shown to be safe if consumed at dietary levels, as there has been no reports of adverse events in humans when consumed as part of the diet [56]. Therefore, daily MBE intake before the onset of bone mass decline may be helpful for bone maintenance, through exerting an antioxidative action.

## 5. Conclusions

In conclusion, MBE protects tyrosine residues of I-κB from nitration, thereby inhibiting NF-κB transnucleation, which results in the restoration of OB differentiation, whereas MBE inhibits OC differentiation and function. Furthermore, the oral administration of MBE improves bone metabolism through inhibiting bone resorption and stimulating bone formation in osteopenic mouse models.

## Figures and Tables

**Table 1:** Specific primer sets.

Genes	Proteins (Abbreviations)	Primer Sequences		Product (bp)	Accession Numbers
*Bmp2*	Bone morphogenetic protein 2 (BMP2)	Forward:	5′-TGA CTG GAT CGT GGC ACC TC-3′	112	NM_007553.3
		Reverse:	5′-CAG AGT CTG CAC TAT GGC ATG GTT A-3′		
*Bmp4*	Bone morphogenetic protein 4 (BMP4)	Forward:	5′-AGC CGA GCC AAC ACT GTG AG-3′	68	NM_007554.2
		Reverse:	5′-TCA CTG GTC CCT GGG ATG TTC-3′		
*Runx2*	Runt-related transcription factor 2 (RUNX2)	Forward:	5′-TCA CTA CCA GCC ACC GAG A-3′	81	NM_001145920.2
		Reverse:	5′-CTG CTT GCA GCC TTA AAT ATT CC-3′		
*Osx*	Osterix (OSX)	Forward:	5′-GTC CTC TCT GCT TGA GGA AGA A-3′	79	NM_130458.4
		Reverse:	5′- GCC AAA TTT GCT GCA GGC T-3′		
*Mepe*	Matrix extracellular phosphoglycoprotein (MEPE)	Forward:	5′-ATG CAG GGA GAG CTG GTT AC-3′	84	NM_053172.2
		Reverse:	5′-TGG TTC CCT TTG GAC TCT TC-3′		
*Ocn*	Osteocalcin (OCN)	Forward:	5′-GTG AGC TTA ACC CTG CTT GT-3′	96	NM_130458.4
		Reverse:	5′-AGC ACA GGT CCT AAA TAG TGA TAC C-3′		
*Actb*	β-actin	Forward:	5′-CAT CCG TAA AGA CCT CTA TGC CAA C-3′	171	NM_007393.5
		Reverse:	5′-ATG GAG CCA CCG ATC CAC A-3′		
*Nfatc1*	Nuclear factor of activated T cells 1 (NFATC1/NFAT2)	Forward:	5′-GCC TCG AAC CCT ATC GAG TG-3′	121	AI449492
		Reverse:	5′- AGT TAT GGC CAG ACA GCA CC-3′		
*Ctsk*	Cathepsin K (CTSK)	Forward:	5′-TAC CCA TAT GTG GGC CAG GA-3′	107	AI323530
		Reverse:	5′- AGT TAT GGC CAG ACA GCA CC-3′		
*Gapdh*	Glyceraldehyde-3-phosphate dehydrogenase (GAPDH)	Forward:	5′- AAC TTT GGC ATT GTG GAA GG -3′	132	NM_008084
		Reverse:	5′- GGA TGC AGG GAT GAT GTT CT -3′		
